# Template-based temporomandibular joint puncturing and access in minimally invasive TMJ surgery (MITMJS) – a technical note and first clinical results

**DOI:** 10.1186/s13005-019-0194-8

**Published:** 2019-04-02

**Authors:** Matthias Krause, Hans Martin Dörfler, Daniel Kruber, Heike Hümpfner-Hierl, Thomas Hierl

**Affiliations:** 1 Department of Oral & Maxillofacial Plastic Surgery, Leipzig University, Liebigstr. 12, 04103 Leipzig, Germany; 2 Faculty of Mechanical and Energy Engineering, University of Applied Sciences (HTWK), Karl-Liebknecht Str. 145, 04277 Leipzig, Germany; 3 Department of Oral and Maxillofacial Plastic Surgery, Helios Vogtlandklinikum Plauen, Roentgenstr. 2, 08529 Plauen, Germany

**Keywords:** Surgical template, Computer-guided surgery minimally invasive temporomandibular joint surgery

## Abstract

**Background:**

Minimally invasive temporomandibular joint surgery (MITMJS) is an option for patients suffering from severe internal derangement or adhesions. To improve TMJ access, a workflow to create surgical templates is introduced.

**Methods:**

A workflow to generate a dividable patient specific template based on CBCT and optical scanning to access the joint is introduced. In a first clinical trial 3 patients (6 joints) were treated by way of template-guided endoscopically-assisted TMJ therapy (3 arthrocenteses and 3 arthroscopies).

**Results:**

Generation and clinical use of the templates was as planned. All templates showed perfect fit and permitted instant access to the TMJ. All surgeries were performed without complications.

**Conclusions:**

Template-guidance could improve the feasibility of endoscopically-assisted TMJ therapy. An important issue is the capability to dis- and remount the template during surgery. Using in-house production, costs are affordable.

**Trial registration:**

This study was registered at the Ethic Committee of the Berlin Medical Chamber (Eth-30/17, 12/06/2017).

## Background

Minimally invasive temporomandibular joint surgery (MITMJS) like arthrocentesis or arthroscopy has been successfully used in the treatment of internal derangement (ID) of the temporomandibular joint (TMJ) and was first described by Onishi [1], who also reported the use of the arthroscope for diagnostics. In 1982, Muracami and Hoshino [2] developed the nomenclature of arthroscopic anatomy.

MITMJS seems to be connected with a relatively low complication rate less than 1.5% [3]. Although bleeding within the superior TMJ space was observed in 8.5% of the arthroscopies [4], it was not severe in any of the cases and was not considered as a real complication. It is, however, desirable to reduce bleeding which could be achieved using new techniques.

Computer-assisted arthroscopy is a promising technology to decrease complication rates and operation time for MITMJS [5]. Computer-assisted design (CAD) and computer-assisted manufacturing (CAM) procedures combined with three-dimensional printing techniques facilitate the use of surgical templates and are well described in craniomaxillofacial surgery [6–9].

In this technical report, a novel workflow to design and generate a surgical template via CAD/CAM to gain access to the TMJ is introduced. It should assist minimally invasive temporomandibular joint arthrocentesis or arthroscopy to combine the potential advantages of these novel techniques and first clinical results are presented. This is the first known report of template-guided therapy in the TMJ other than the use of a cutting guide for tumor resection [10] and one of the first considering joint surgery besides arthroplasty resection guides [11].

## Material and methods

### Template generation

The workflow to generate a surgical template via CAD/CAM is fully digitally.

The preoperative 3D planning procedure is performed after obtaining a cone beam computed tomography (CBCT) of the skull, an optical facial scan and using a planning software (Facial Analysis Tool: FAT) [12, 13].

Thus 4 steps are required. The first step is the CBCT (Kodak 9500 3D, Carestream Health, Toulouse, France). It is used for TMJ diagnosis and provides volumetric images of the anatomic structures of the patients’ craniofacial skeleton. Alternatively CT or MRI data could be used. If CT or CBCT was to be used, the orbits should be not included due to unnecessary radiation exposure. The second step is optical scanning of the face (Vectra® M3, Canfield Scientific Inc., Fairfield, NJ, USA). It allows a fast, radiation free and precise generation of a detailed 3D surface mesh of the face. The optical scan adds information to the CBCT as it shows areas of hair-bearing skin which will not be included in the template and adds realism as the texture of the skin is shown.

**Fig. 1:**
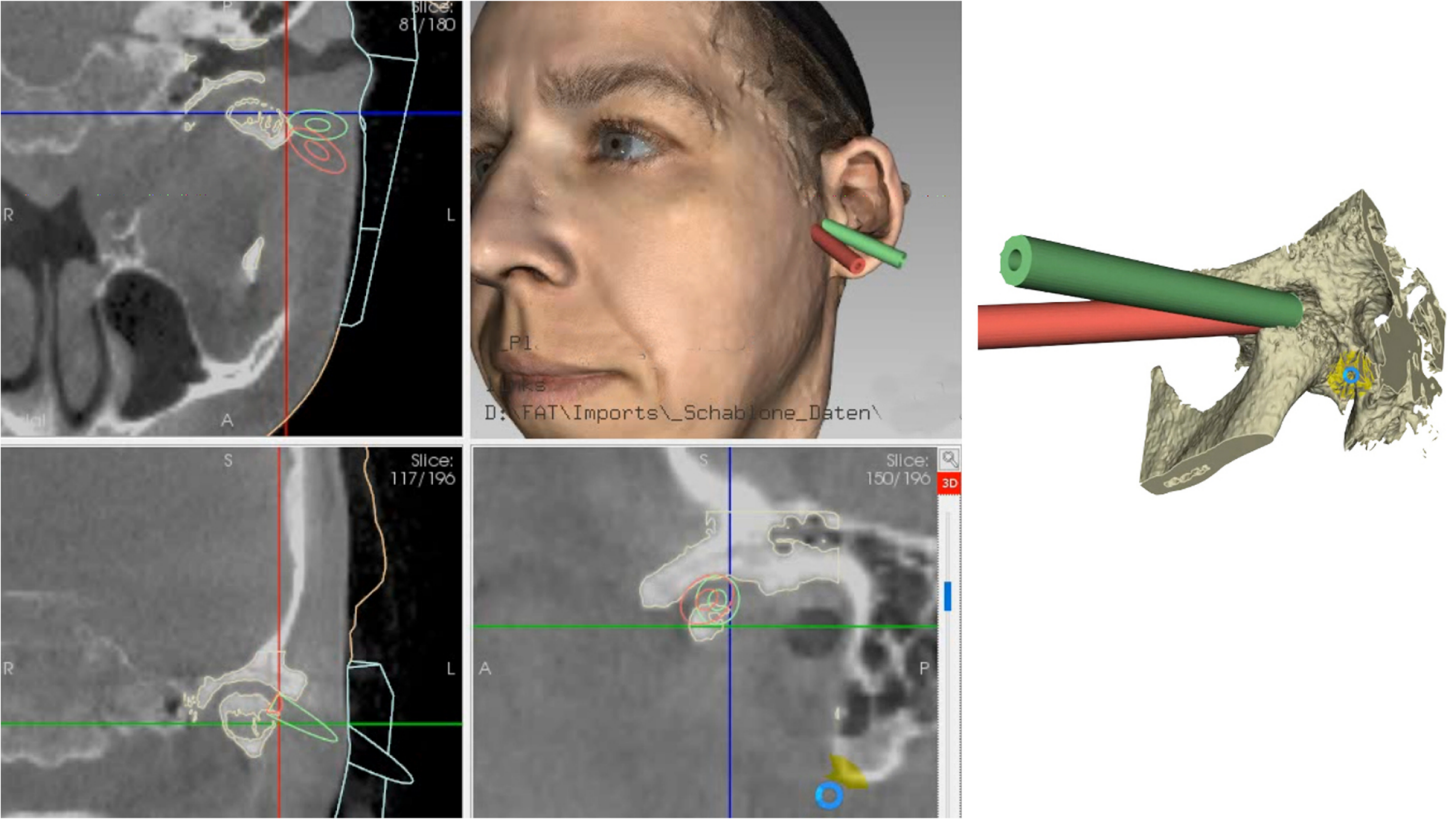
Planning of the pilot channels in FAT software

**Fig. 2:**
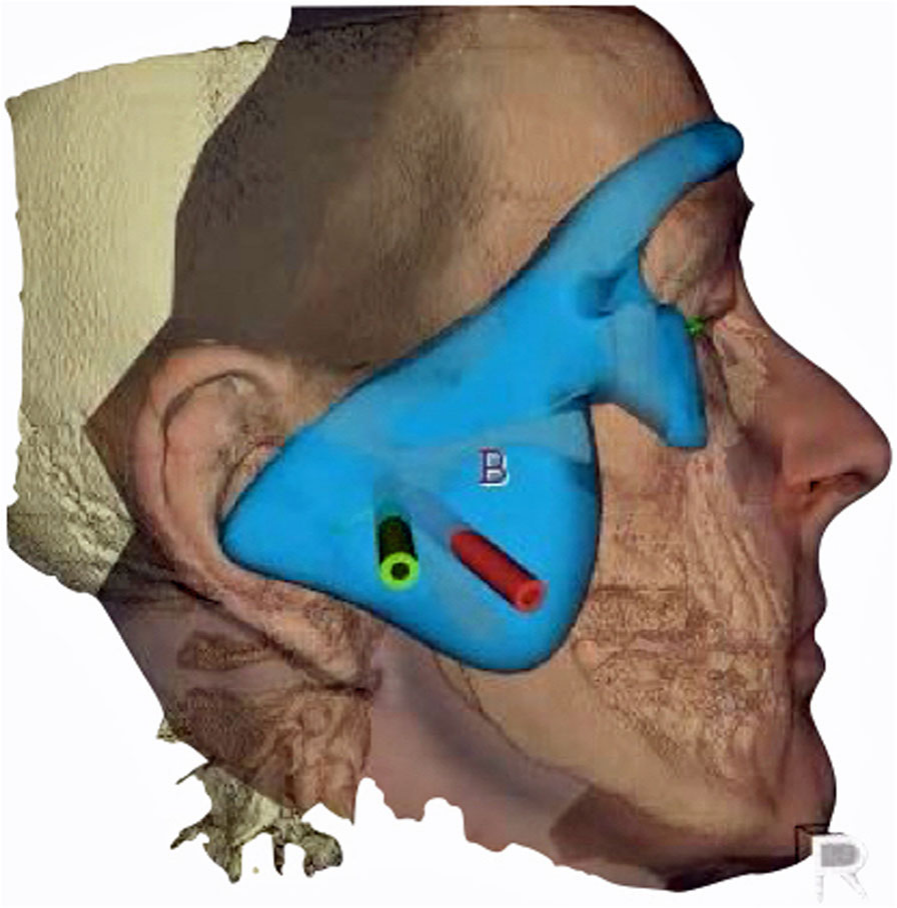
Surgical template prior to separation in 2 parts. The extensions to guarantee fit and the working channels are visible. Overlay of CBCT and optical scan

Next step is the conversion of the DICOM data to surface data which is matched with the optical scan data of the face using the iterative closest point algorithm in FAT. Now is it possible to plan e. g. two working channels for the endoscope and the manipulation instruments according to the given anatomy, pathology, and the instrument dimensions following the described access by Muracami and Hoshino [2]. The length of the channels can be tailored according to the working length of the endoscope to ensure the exact position. As soon as the pilot channels have been placed, the template can be designed in FAT. The template has two extensions to the forehead and cheek and a centering plug to the cavum conchae to guarantee its exact position (Figs. 1 and 2). Thus the template acts as a puncturing aid of the skin and directs the endoscope to the desired spot within the TMJ. It is important to integrate the desired surgical plan in this stage in the template, i.e. choosing a joint entrance spot which will allow all later movements of the endoscope or further working channels. Thus an entrance into the TMJ capsule ranging from anterior to posterior can be planned.

**Fig. 3:**
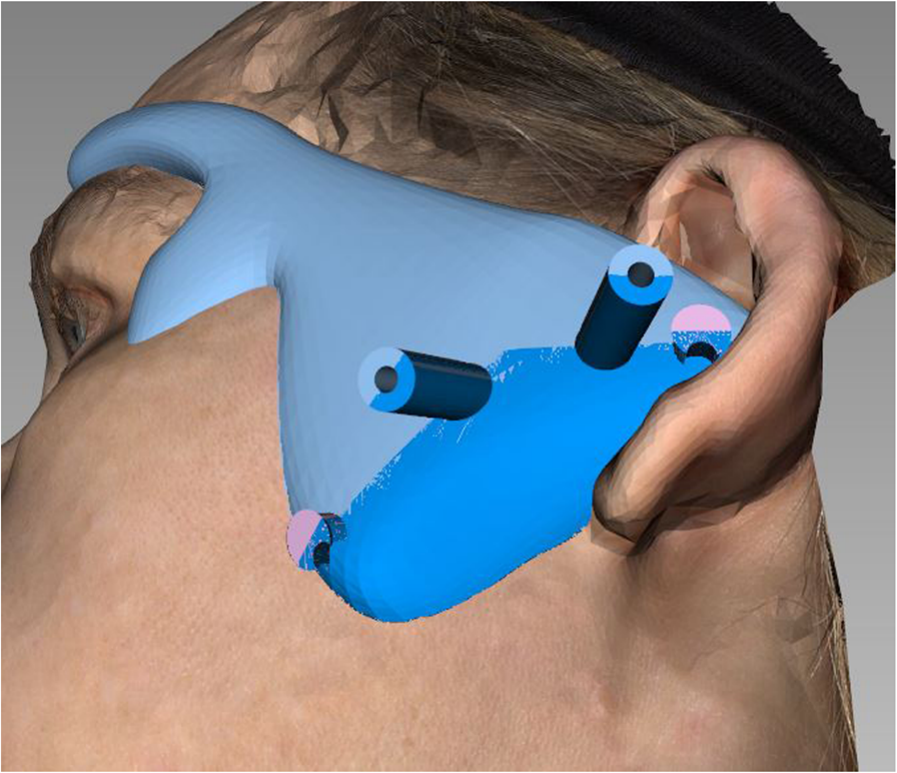
Template is split along the working channels to allow removal during surgery. Inferior and superior to the working channels two posts featuring an undercut are seen which are needed to connect the two parts firmly during surgery using heavy elastics or wire

**Fig. 4:**
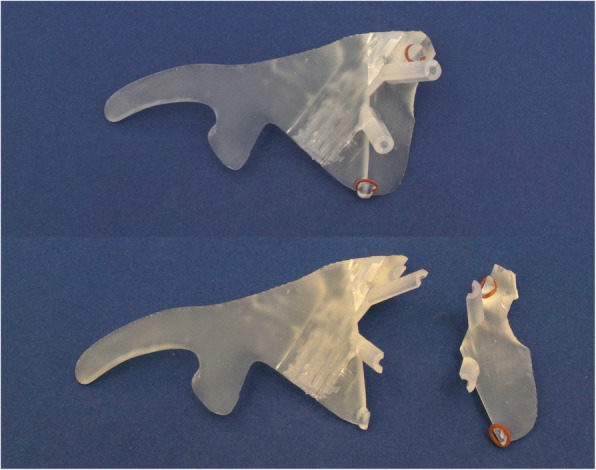
The printed template. It is clear to see a potential imperfect fit, resp. intraoperative bleeding

Furthermore, the template is separated in two parts along the working channels so it may be removed and repositioned during surgery. Therefore it is possible to change any time from template-guided MITMJS to a conventional procedure without interference by the template in the further course of MITMJS (Figs. 3 and 4), or to reset the endoscope position by repositioning the template if the orientation is problematic.

The fourth and last step is printing the surgical template (Formlab 2; Formlabs Inc., Somerville, USA) using a CE-certified clear biocompatible photopolymer resin (Dental SG resin) which is a durable transparent hard plastic that can be sterilized (Fig.4).

### Surgical procedure

**Fig. 5:**
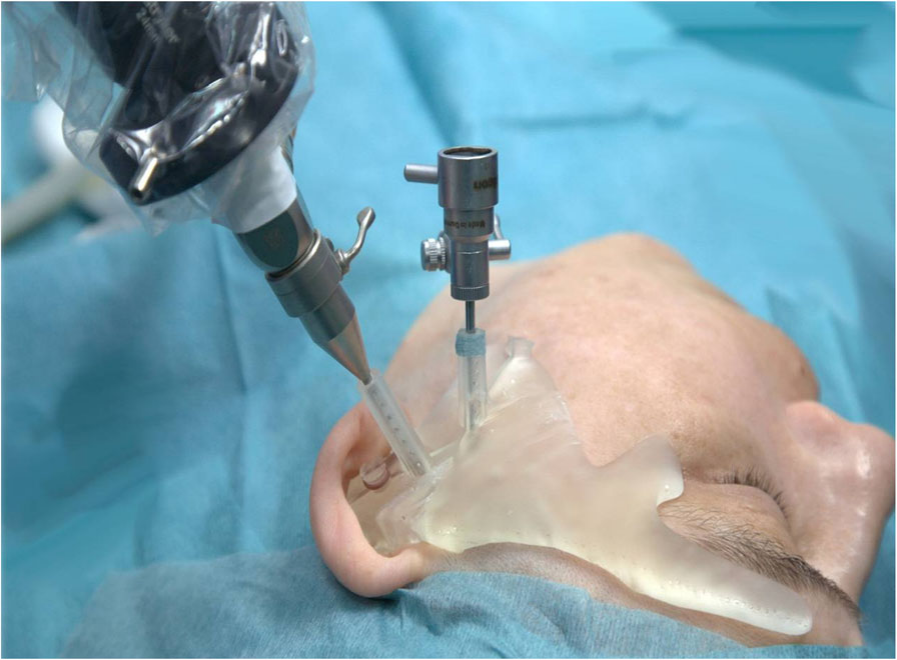
Intraoperative view after insertion of the protecting sheats and the endoscope

Prior to surgery the fit of the template is checked on the awake patient. In sedation or general anesthesia, the template is positioned and a trocar is inserted in the working channels to mark the skin. Next a stab incision is performed after temporary removal of the guide at the skin markings and the subcutaneous tissue is slightly spread with fine scissors. After the guide has been repositioned, the trocar is reinserted and advanced. Then the protecting sheaths are introduced which will guide the endoscope and the manipulation instruments. Now the video-assisted surgical procedure starts. If required the template may be removed or repositioned if an instrument dislocation occurs at any time (Fig. 5).

### Patients and procedures

MITMS using the surgical template was performed from July 2017–April 2018 in three patients with a total number of 6 procedures in 6 joints: three arthroscopies and three arthrocenteses. Table 1 shows the relevant patient data.

**Table 1:** Patient data and performed surgical procedures (*n* = 3)

no	gender	age	diagnosis	site	procedure
1	f	53	ID, Ws V	l	asc, lv, iaj 10 mg TriamHEXAL® (HEXAL AG)
					asc, lv, iaj 10 mg TriamHEXAL® (HEXAL AG)
2	f	44	ID, Ws V	r	asc, lv, iaj 10 mg TriamHEXAL® (HEXAL AG)
3	m	75	ID, Ws IV	r	ac, lv, iaj 1,5 ml Hya Ject® (ORMED GmbH)
					ac, lv, iaj 1,5 ml Hya Ject® (ORMED GmbH)
					ac, lv, iaj 1,5 ml Hya Ject® (ORMED GmbH)

Stated are the number of patients, gender, age, diagnosis, site and procedure. *ID* internal derangement, *WS* Wilkes stage (I-V), *asc* arthroscopy, *ac* arthrocentesis, *lv* lavage, *iaj* intra articulary injection

The mean age was 57.3 years (range 44–77 ys), the group consisted of two women and one man suffering from internal derangement (ID) of the TMJ. According to the classification for ID of the TMJ proposed by Wilkes [3] two patients suffered from Wilkes stage V and one patient suffered from Wilkes stage IV [14].

The MITMS procedures included three arthroscopies in two patients and three arthrocenteses in one patient. In three times the left and in three times the right joint were treated by 6 lavages followed by 6 injections (10 mg Triamcinolonacetonid, TriamHEXAL®, HEXAL AG, Holzkirchen, Germany or Hyaluronic acid, Hya Ject®, ORMED GmbH, Freiburg, Germany).

This study was approved by the Local Ethics Committee. This report followed the Declaration of Helsinki on medical protocols and ethics.

## Results

All templates could be generated as planned in an in-house workflow. Planning time decreased from three to about 2 hours in the last case. Printing time lasted about 7 h and the costs of the used CE certified resin ranged at 5–6 € per template. The fit of all templates was judged excellent and assisted in all temporomandibular procedures well. Instrument placement was quickly performed as planned with direct access to the respective regions. All surgeries were without complications (no bleeding, infection, nerve injury).

The average arthocenthesis required 10–15 min operation time, arthroscopies lasted between 70 and 105 min. Follow-up ranged from 3 to 6 months with a pain reduction from preoperatively 6–8 to postoperatively 2–3 on a 10 point visual analogue scale (10: maximum pain, 0: no pain).

## Discussion

Template-guiding has been applied to many procedures in craniomaxillofacial surgery ranging from implant insertion and preparing of free fibula flaps to orthognathic procedures. This report adds a new mosaic piece in several respects. It introduces templates to TMJ-therapy and, in contrast to most templates, is designed in a modular way allowing its disassembly, removal, and refixation during surgery. This is different from an implant guide, where the drill and implant are inserted through a fixed guide which may not be removed as long as the instrument is in situ.

Regarding TMJ procedures, this novel approach allows the exact planning of the endoscope and manipulation instruments position which might make TMJ procedures easier and safer for novices in the field. The introduced template should assist the surgeon in several ways: first it allows easy puncturing at the correct location. During surgery it can be removed, but if the surgeon should be insecure about the exact position, it may be repositioned to direct the endoscope to its initial location. Thus the template aids in the difficult triangulation-orientation during the surgical procedure.

To gain access to the TMJ in endoscopically-assisted surgery, the mandible is manipulated (e.g. protracted, distracted, jaw opened or closed) during puncturing. As this is a technical note regarding first results, integration of manipulations into the design of the template has not been performed by now. However, dental splints (e.g. to distract – protrude the mandible) could be inserted during scanning and repositioned at surgery. In these cases MRI scans should be used to prevent undue radiation exposure. Furthermore, the modular design could be changed to include different angulations of the guiding channels during surgery (e.g. mouth opened or closed) to increase the value of the template.

The data derived from this report cannot prove the assumption that MITMJS will benefit from template-guidance by now, thus further studies are needed. The presented promising data, however, will encourage our use of template guidance in TMJ procedures. The above mentioned additions show, that much more planning might be integrated into the template design. Time sparing and safety will be questions of future investigations. The modular approach will permit adaptions to many different procedures in TMJ-therapy e. g. with more than two working channels. In this report CBCT and optical scanning were combined. Depending on the situation, our workflow could also use MRI data, which, however, were not used in our patients. The use of optical scanning is not mandatory but is interesting in two respects: it demonstrates the combination of multimodal 3D-data fusion and in our cases eases the template design, as areas of hair-bearing skin, which could interfere with perfect template fit are spared. As an alternative design of the template with no „ear-plug “and two extensions, an intraoral maxillary occlusal wafer connected to the extraoral template could be used. Then - like in our workflow - multimodal data like an optical maxillary dental cast scan registered to the CBCT would be appropriate.

An important issue are costs, working time and legal concerns. Using an in-house workflow the singular price is acceptable, but planning demands a high amount of time which could be cut down if more experience was gained. According to the report of Otero et al. [15] European legislation permits the in-house creation of templates, if certified materials are used like in this study. If this applies to non-EC countries, too, is unknown to the authors. Using the described workflow a typical template will have material costs of about 5–6 €. Regarding time needed to design the template, the utilized software and the training of the surgeon are critical aspects. In our first cases about 3–2 h were needed per case. Further studies will show if this might be speeded up and 1 h seems realistic using our software workflow. Furthermore, software improvements should allow additional time gain. Finally the costs regarding data acquisition have to be considered, but as in our patients the data used for TMJ diagnostics could be used, no additional costs were necessary. As the template is intricately combined with the surgical procedure, designing the template by a third party to minimise time effort, however, seems questionable.

Although this technical note focuses on minimally invasive TMJ procedures, the workflow regarding dismountable puncturing or guiding templates may be used for further applications in TMJ, CMF, or neurosurgery.

## Conclusion

Our feasibility report on template-guided MITMJS shows a promising new application of templates in the field of medicine. It is the first report regarding arthroscopy or endoscopically-assisted joint procedures known to the authors. Using an in-house workflow, template construction is affordable, although planning is still time consuming. If the use of templates will improve surgical outcome – especially in the hands of novices - remains unclear by now and will be investigated in future studies. As our modular design permits easy changing from template-guiding to free-hand surgery and vice-versa, at least no adverse effects are expected.

## Abbreviations

CBCT: Cone beam computer tomography
CT: Computer tomography
MITMJS: Minimal invasive temporo-mandibular joint surgery
MRI: Magnetic resonance imaging
TMJ: Temporo-mandibular joint
