# Correction to: Template-based temporomandibular joint puncturing and access in minimally invasive TMJ surgery (MITMJS) – a technical note and first clinical results

**DOI:** 10.1186/s13005-019-0197-5

**Published:** 2019-06-18

**Authors:** Matthias Krause, Hans Martin Dörfler, Daniel Kruber, Heike Hümpfner-Hierl, Thomas Hierl

**Affiliations:** 1 Department of Oral & Maxillofacial Plastic Surgery, Leipzig University, Liebigstr. 12, 04103 Leipzig, Germany; 2 Faculty of Mechanical and Energy Engineering, University of Applied Sciences (HTWK), Karl-Liebknecht Str. 145, 04277 Leipzig, Germany; 3 Department of Oral and Maxillofacial Plastic Surgery, Helios Vogtlandklinikum Plauen, Roentgenstr. 2, 08529 Plauen, Germany

**Correction to: Head Face Med**

**https://doi.org/10.1186/s13005-019-0194-8**

Following publication of the original article [1], the authors reported that the registration number of the ethics approval was accidentally entered under trial registration. A trial registration was not performed by the authors.

## References

[1] Krause M, Dörfler HM, Kruber D, Hümpfner-Hierl H, Hierl T. Template-based temporomandibular joint puncturing and access in minimally invasive TMJ surgery (MITMJS) – a technical note and first clinical results. Head Face Med. 10.1186/s13005-019-0194-8. 10.1186/s13005-019-0194-8 PMC6444874 30940211

